# DrugBank 4.0: shedding new light on drug metabolism

**DOI:** 10.1093/nar/gkt1068

**Published:** 2013-11-06

**Authors:** Vivian Law, Craig Knox, Yannick Djoumbou, Tim Jewison, An Chi Guo, Yifeng Liu, Adam Maciejewski, David Arndt, Michael Wilson, Vanessa Neveu, Alexandra Tang, Geraldine Gabriel, Carol Ly, Sakina Adamjee, Zerihun T. Dame, Beomsoo Han, You Zhou, David S. Wishart

**Affiliations:** ^1^Department of Computing Science, University of Alberta, Edmonton, AB, Canada T6G 2E8, ^2^Department Biological Sciences, University of Alberta, Edmonton, AB, Canada T6G 2E8, ^3^Faculty of Pharmacy and Pharmaceutical Sciences, University of Alberta, Edmonton, AB, Canada T6G 2N8 and ^4^National Institute for Nanotechnology, 11421 Saskatchewan Drive, Edmonton, AB, Canada T6G 2M9

## Abstract

DrugBank (http://www.drugbank.ca) is a comprehensive online database containing extensive biochemical and pharmacological information about drugs, their mechanisms and their targets. Since it was first described in 2006, DrugBank has rapidly evolved, both in response to user requests and in response to changing trends in drug research and development. Previous versions of DrugBank have been widely used to facilitate drug and *in silico* drug target discovery. The latest update, DrugBank 4.0, has been further expanded to contain data on drug metabolism, absorption, distribution, metabolism, excretion and toxicity (ADMET) and other kinds of quantitative structure activity relationships (QSAR) information. These enhancements are intended to facilitate research in xenobiotic metabolism (both prediction and characterization), pharmacokinetics, pharmacodynamics and drug design/discovery. For this release, >1200 drug metabolites (including their structures, names, activity, abundance and other detailed data) have been added along with >1300 drug metabolism reactions (including metabolizing enzymes and reaction types) and dozens of drug metabolism pathways. Another 30 predicted or measured ADMET parameters have been added to each DrugCard, bringing the average number of quantitative ADMET values for Food and Drug Administration-approved drugs close to 40. Referential nuclear magnetic resonance and MS spectra have been added for almost 400 drugs as well as spectral and mass matching tools to facilitate compound identification. This expanded collection of drug information is complemented by a number of new or improved search tools, including one that provides a simple analyses of drug–target, –enzyme and –transporter associations to provide insight on drug–drug interactions.

## INTRODUCTION

DrugBank is a comprehensive repository of drug, drug–target and drug action information developed, maintained and enhanced by extensive literature surveys performed by domain-specific experts and skilled biocurators. The quality, breadth and uniqueness of its data have made DrugBank particularly popular (>8 million web hits/year) and highly regarded among pharmaceutical researchers, medicinal chemists, clinicians, educators and the general public. Because most of the data in DrugBank are expertly curated from primary literature sources, it has become the referential drug data source for a number of well-known databases such as PharmGKB (1), ChEBI (2), KEGG (3), GeneCards (4), PDB (5), PubChem (6), UniProt (7) and Wikipedia. Since its first release in 2006, DrugBank has been continuously evolving to meet the growing demands of its users and the changing needs of its rapidly expanding user base. The first version of DrugBank was limited to providing data on selected Food and Drug Administration (FDA)-approved drugs and their drug targets (8). Pharmacological, pharmacogenomic and molecular biological data were added to DrugBank 2.0, along with a significant increase in the number of approved and experimental drugs (9). DrugBank 3.0, released in 2010, was expanded to include data on drug–drug and drug–food interactions, metabolic enzymes and transporters as well as pharmacokinetic and pharmacoeconomic information (10). For 2014, DrugBank has been enhanced to capture the increasing body of quantitative knowledge about drugs and improved technologies to detect drugs, their metabolites and their downstream effects. In particular, significant improvements and large-scale additions in the areas of QSAR (quantitative structure activity relationships), ADMET (absorption, distribution, metabolism, excretion and toxicity), pharmacometabolomics and pharmacogenomics have been made. Existing information about drug structures, drug salt-forms, drug names, drug targets and drug actions has also been expanded and updated. Numerous approved and experimental drugs have been added along with a number of new data fields describing each drug. New search tools have also been developed or improved on to increase the ease with which information can be found.

Many of the enhancements made over the past 3 years were stimulated by user feedback and suggestions. We are grateful to our users and continue to strive to meet their needs. Further details on the additions and enhancements made to DrugBank 4.0 are described later.

## DATABASE ADDITIONS AND ENHANCEMENTS

The development and evolution of DrugBank, including previous data content additions, curation protocols, quality control methods, general layout, interface features and data sources, has been described previously (8–10). Here we shall focus on enhancements and changes made since the release of DrugBank 3.0. In particular, we will discuss (i) enhancements made to existing data, (ii) the addition of new data fields, (iii) new and enhanced search features and (iv) a new user feedback/commenting system.

### Enhancement of existing data

DrugBank has evolved significantly since its inception in 2006 with a progressive expansion in the depth and breadth of its data as well as improvements to the quality and reliability of its information. The enhancements and additions in data content between DrugBank 1.0, 2.0, 3.0 and 4.0 are summarized in Table 1. As seen in this table, DrugBank 4.0 has seen a further 40% increase in the number of data fields per DrugCard. The number of FDA-approved drugs has increased by 19%, biotech drugs by 14% and >1200 new investigational drugs (in phase I, II or III trials) have been added. Likewise, the number of drugs with transporters and carrier proteins has increased by 40 and 30%, respectively. Additionally, the number of QSAR parameters has grown by 64% and the number of ADMET parameters by 650%. The number of drug–drug interactions and the number of drug–food interactions have grown by 2.5% and 14%, respectively. Similarly, the number of drug target single-nucleotide polymorphisms (SNPs) has grown by 10%, whereas the number of SNPs associated with adverse drug effects (SNP-FX and SNP-ADR) has grown by 78%. In addition, the number of illustrated drug action pathways has grown by 38%. Furthermore, chemical synthesis information has been added to >1200 drugs, with many synthesis references now hyperlinked to patents and reaction schemes maintained by Drugsyn.org (http://www.drugsyn.org).

**Table 1.** Comparison between the coverage in DrugBank 1.0, 2.0, 3.0 and DrugBank 4.0

Category	1.0	2.0	3.0	4.0
Number of data fields per DrugCard	88	108	148	208
Number of search types	8	12	16	18
Number of illustrated drug-action pathways	0	0	168	232
Number of drugs with metabolizing enzyme data	0	0	762	1037
Number of drug metabolites with structures^a^	0	0	0	1239
Number of drug-metabolism reactions^a^	0	0	0	1308
Number of illustrated drug metabolism pathways^a^	0	0	0	53
Number of drugs with drug transporter data	0	0	516	623
Number of drugs with taxonomic classification information^a^	0	0	0	6713
Number of SNP-associated drug effects	0	0	113	201
Number of drugs with patent/pricing/manufacturer data	0	0	1208	1450
Number of food–drug interactions	0	714	1039	1180
Number of drug–drug interactions	0	13 242	13 795	14 150
Number of biochemical target-mediated drug–drug interactions^a^	0	0	0	35 632
Number of biochemical enzyme-mediated drug–drug interactions^a^	0	0	0	226 463
Number of biochemical transporter-mediated drug–drug interactions^a^	0	0	0	60 644
Number of biochemical drug–drug interactions (all)^a^	0	0	0	322 739
Number of ADMET parameters (Caco-2, LogS)	0	276	890	6667
Number of QSAR parameters per drug	5	6	14	23
Number of drugs with drug–target binding constant data^a^	0	0	0	791
Number of drugs with NMR spectra^a^	0	0	0	306
Number of drugs with MS spectra^a^	0	0	0	384
Number of drugs with chemical synthesis information	0	38	38	1285
Number of FDA-approved small molecule drugs	841	1344	1424	1552
Number of FDA small molecule drugs with salt-form structures^a^	0	0	0	474
Number of biotech drugs	113	123	132	284
Number of nutraceutical drugs	61	69	82	87
Number of withdrawn drugs	0	57	68	78
Number of illicit drugs	0	188	189	190
Number of experimental drugs	2894	3116	5210	6009
Number of investigational drugs (phase I, II and III trials)^a^	0	0	0	1219
Total number of experimental and FDA small molecule drugs	3796	4774	6684	7561
Total number of experimental and FDA drugs (all types)	3909	4897	6816	7713
Number of all drug targets (unique)	2133	3037	4326	4115
Number of approved-drug enzymes/carriers (unique)	0	0	164	245
Number of all drug enzymes/carriers (unique)	0	0	169	253
Number of external database links	12	18	31	33

^a^New data.

Data quality and currency have always been two of the top priorities for the DrugBank curation team. Consequently, significant efforts over the past 3 years have been directed at improving the quality of existing DrugCards and keeping them as up-to-date as possible. Unlike DrugBank (i.e. the database), which has a 2–3-year release cycle, DrugBank’s DrugCards are continuously updated as new information becomes available. Because new drugs are constantly being approved, and old drugs are perpetually being re-characterized, re-purposed or removed, our curation team is continuously combing the literature to update existing DrugCards or to add new DrugCards. As a result, hundreds of drug descriptions, drug targets, indications, pharmacodynamics data, mechanisms of action and metabolism data fields have been added, re-written and/or expanded over the past 3 years. We have also updated and re-written all of DrugBank’s drug–drug interactions. In particular, we have included or updated all interactions where modifications in drug therapy are recommended either by the manufacturer or other data sources.

Drug nomenclature is particularly important for pharmacists, physicians and the drug industry. Many drugs have dozens or even hundreds of alternate names, brand names or synonyms, often arising because of various trademark, patenting and/or marketing requirements. For the latest release of DrugBank, all of its synonyms and brand names were extensively reviewed. Lengthy chemical and salt-form names were removed and only the most common synonyms have been included, enabling users to more easily browse alternate drug names. Brand names or synonyms appearing on more than one DrugCard were also identified and edited or removed as required. In total, >8000 duplicated or partially duplicated brand names and synonyms were edited or removed as part of DrugBank 4.0’s nomenclature remediation effort.

DrugBank’s experimental drug data set was also reviewed, remediated and expanded. In particular, experimental drugs binding to non–human targets were removed with the exception of those that bind to known bacterial, viral and fungal pathogens. Experimental drug targets originally referenced to the RCSB Protein Data Bank (5) are now referenced to the original publications. Additional experimental drugs were added from a variety of literature sources and online databases, such as ChEMBL (11). Further data quality checks on drug structures were carried out by identifying InChI keys and ChEBI IDs appearing on more than one DrugCard. In some cases, duplicate IDs were the result of duplicate DrugCards, whereas in other cases, the IDs referred to stereoisomers that did not have stereochemistry depicted in their structures. These structures were manually corrected. In total, ∼600 corrections of various forms were performed as part of the DrugBank 4.0 structure remediation effort.

Drug classes are a useful way of grouping drugs with similar therapeutic effects and mechanisms of action. In the latest version of DrugBank, we expanded on the information provided by the Anatomical Therapeutic Chemical (ATC) Classification System defined by the WHO Collaborating Centre for Drug Statistics Methodology (12). The first four levels of the classification system (anatomical main group, therapeutic subgroup, pharmacological subgroup and chemical subgroup) are listed for each drug with links to what we call CategoryCards. The CategoryCard for each anatomical main group shows a listing of therapeutic subgroups, which can be expanded to show pharmacological subgroups, which can be further expanded to show chemical subgroups, which are expandable to finally show the fifth level, chemical substances or drug names. This new tree structure and the new browsing function called Category Browse effectively replaces the old PharmaBrowse found in earlier versions of DrugBank. Adopting this well-established classification system in DrugBank 4.0 enables users to more easily search for drugs with similar pharmacologic effects.

### New data fields

As noted earlier, significant efforts for this year’s release of DrugBank have been directed toward adding new data on drug metabolism, ADMET, pharmacometabolomics, pharmacogenomics and a variety of QSAR. New data fields are also highlighted in Table 1. These additions to DrugBank 4.0 were motivated by the fast-moving developments in the fields of xenobiotic metabolism, pharmacometabolomics, pharmacogenomics, pre-clinical drug screening and computer-aided drug design.

With regard to DrugBank 4.0’s effort to capture more drug metabolism information, it is important to note that the database now contains >1200 drug metabolites (including their structures, names, activity, abundance and other detailed data) along with drug metabolism reaction data for >1000 approved drugs. These drug metabolism data include the sequence of reactions, reaction types and whether drug metabolites are active, inactive and/or major circulating entities. In addition, DrugBank now offers comprehensive, colorful, manually drawn interactive drug metabolism pathways [using the SMPDB pathway model (13)] for >50 drugs. We believe that the addition of large numbers of drug metabolites to DrugBank should also assist metabolomic and pharmacometabolomic studies by facilitating the identification of xenobiotics in biological samples (via MS spectrometry). By providing the drug research community with >1200 drug metabolites (along with their corresponding phase I and II enzymes), we are also hoping that new and improved software could be developed for predicting the structure of drug metabolites. Efforts are already underway in our laboratory to develop open access freeware for drug metabolism prediction.

In addition to the substantial additions to DrugBank’s drug metabolism data, the quantity and diversity of ADMET data in DrugBank 4.0 has also been significantly increased. Although a modest amount of ADMET data was included in previous versions of DrugBank (8–10), it was generally limited to a small number of approved drugs. However, by taking advantage of several recently released software packages for predicting ADMET properties (14) and mining newly published ADMET surveys (15,16), it has been possible to add ∼30 more ADMET data fields to nearly all of DrugBank’s drug entries. These include measured and/or predicted ADMET values for Caco cell permeability (as a proxy for intestinal permeability), blood–brain barrier permeability, human intestinal absorption levels, P-glycoprotein activity, Cyp450 substrate preferences, renal transport activity, carcinogenicity, toxicity, Ames test activity and human Ether a-go-go Related Gene (hERG) activity, along with their probability scores.

As the fields of metabolomics and pharmacometabolomics continue to evolve, we believe there is an increasing need to develop drug databases that are compatible with the needs of metabolomic researchers. In particular, referential mass spectrometry (MS) and nuclear magnetic resonance (NMR) spectra of pure drugs and their metabolites are particularly important for identifying and/or quantifying compounds in biological matrices. Prescription and over-the-counter medications are frequently found in human biofluid samples characterized in metabolomic studies. To meet these compound identification/characterization needs, >750 NMR and 3763 MS reference spectra for ∼400 different drugs have been added to DrugBank 4.0. Many of these spectra were experimentally acquired by our laboratory (17), whereas others have been assembled from freely available online sources (18,19). In addition to including these reference MS and NMR spectra, we have also added a number of advanced spectral viewing and spectral/mass matching tools [originally developed for the HMDB (17)] to facilitate metabolomic studies. Using these tools, users can submit chemical shift or m/z (mass to charge ratio) lists and search against DrugBank’s spectral library for exact or even approximate matches. Users can also browse, zoom and view all of DrugBank’s spectral data to visually compare results and assess spectral matches. To further enhance DrugBank’s compatibility with metabolomic activities, we have also added an extensive chemical taxonomy or chemical classification system that uses the same hierarchical design and nomenclature as developed for other metabolomic databases (17,20). In particular, compounds are classified into ‘Kingdoms’, ‘Superclasses’, ‘Classes’, ‘Subclasses’, ‘Direct Parents’, ‘Molecular Framework’, ‘Substituents’ and ‘Cross-References’ using well-defined rules based on their structural features and structure similarities. This classification system allows users to identify all benzodiazepines, select all alkaloids or extract all peptides in DrugBank using a simple text query.

To facilitate QSAR studies, we have continued to add to DrugBank’s collection of quantitative drug data. This includes not only more molecular descriptors (number of H-bond donors/acceptors, physiological charge, refractivity, polarizability, polar surface area, etc) but also more data on chemical substituents (see chemical taxonomy, earlier) and extensive quantitative information on drug–ligand binding constants. The binding constant data have been mined from several sources including the primary literature and BindingDB (21). The combination of DrugBank’s existing 2D and 3D structural data along with new molecular descriptor, molecular substituent and binding constant data for thousands of drugs should allow researchers to perform much more sophisticated and extensive QSAR studies.

Many drugs exist in a variety of salt forms, and these salt variants can play a significant role in the efficacy, delivery or indication for certain drugs. Historically, DrugBank has only provided the structure of ‘base’ form of the drug. However, to accommodate the requests of drug formulation experts and pharmacists, we have now added salt-form structures to DrugBank 4.0.

### New and enhanced search features

The quality of a database is determined not only by the quality of its content but also by the ease with which users can access or navigate through its data. In this regard, the DrugBank team has continuously strived to improve the database’s user interface, to enhance its search utilities and to respond to user suggestions. For DrugBank 4.0, we have added two new browsing features to ease the retrieval of information: (i) Reaction Browse and (ii) Category Browse. We have also improved the Multi-Search feature of DrugBank’s Interax (Drug Interaction Lookup system) and streamlined the existing browsing utilities including DrugBrowse, GenoBrowse and Association Browse. A simple tool called BioInteractor for rapidly retrieving and analyzing the biochemical data in DrugBank has also been added. We have also upgraded DrugBank’s text search engine to improve the speed and quality of the search results, and we have further enhanced DrugBank’s Data Extractor to make it easier to use and far more accessible. These new and improved search/browsing features should substantially increase the ease with which users are able to navigate, retrieve and extract information from DrugBank.

**Figure 1.**
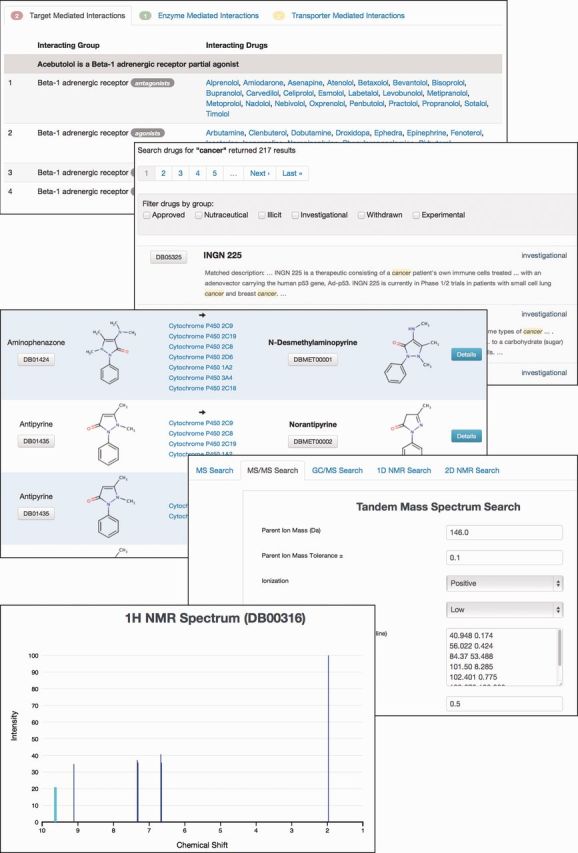
A screenshot montage of some of DrugBank 4.0’s new browsing tools and spectral search features.

Reaction Browse was developed to facilitate the viewing and searching of drug metabolism reactions. Its easily searchable table format allows users to rapidly browse through pages displaying each of the drug metabolism reactions in DrugBank. Each drug, metabolite, enzyme and reaction is hyperlinked to its respective DrugCard, MetaboCard, EnzymeCard and ReactionCard. Search bars above each column of the Reaction Browse table enable users to search by drug name, DrugBank ID, metabolite name, DrugBank metabolite ID, enzyme name, UniProt ID or any combination of the above (Figure 1). Category Browse (which replaces the old PharmaBrowse) is a simple search tool that enables users to browse and search for drugs by category based on pharmacological action or their ATC Classification (12). All four levels of the ATC classification system are listed together to facilitate searching. Each ATC drug category is hyperlinked to a CategoryCard as described earlier. Furthermore, each drug listed in the CategoryCard can be expanded to show drug targets enabling users to easily compare drug targets from the same category (Figure 1).

A new addition to DrugBank’s large selection of search and browsing toolbox is called BioInteractor. BioInteractor uses data on drug-target, -enzyme and -transporter associations to provide insight on drug–drug interactions. It allows users to identify, for instance, which drugs act similarly on the same target. Such drugs may have additive pharmacodynamic effects if given concomitantly. Conversely, two drugs may antagonize the beneficial effects of each other by having opposite actions on a specific drug target. Although clinically relevant drug–drug interactions are well documented in commercial databases such as those produced by Lexi-Comp Inc., the mechanisms of interactions are not always clear. BioInteractor uses ranked drug–enzyme association information to predict enzyme-mediated drug interactions that may be clinically relevant. For this purpose, enzyme inhibitors and inducers associated with DrugBank’s new drug metabolism data have been further categorized as ‘strong’, ‘moderate’ or ‘neither’ based on a set of criteria defined by Bjornsson *et al.* (22). Such a ranking system enables users to differentiate between drug effects on enzymes likely to cause clinically significant changes to the pharmacodynamics of interacting drugs from those unlikely to affect clinical outcomes. A search query with BioInteractor will yield all possible enzyme-mediated interactions, but the ranking system will provide further insight on the probability of a clinically relevant interaction occurring.

In addition to these new search tools, we have also improved DrugBank’s standard search tools. In particular, we have upgraded DrugBank’s text search engine to use Elasticsearch (http://www.elasticsearch.org), an advanced and scalable enterprise search backend. It is also now possible to search across drug targets, drug–drug and drug–food interactions, metabolic reactions, metabolites and other relevant search fields. In addition, search results can now be downloaded using DrugBank’s upgraded Data Extractor. The new Data Extractor appears in all of DrugBank’s standard browse and search windows, allowing users to easily extract, download and save their current data view at any point. In addition, all DrugBank data extractions performed by Data Extractor are saved for up to 1 month, so users can return at any time to retrieve their results. We have also upgraded DrugBank’s Advanced Search, thereby improving the granularity and coverage of data fields in DrugBank. This should allow users to build much more complex queries through Advanced Search’s simple interface. DrugBank’s Advanced Search feature, when combined with the Data Extractor, should allow users to search, retrieve or extract almost any kind of data type or combination of data within DrugBank.

### User feedback, commenting system and news feed

Since the DrugBank was first released in 2006, the web has transformed into a dynamic social environment. News, blogs and scientific online resources provide users with the ability to leave comments and discuss articles. In response to these changes, we have added a commenting feature at the end of each DrugCard, enabling users to submit comments that are moderated by the DrugBank curation team. This commenting system was designed to enable users to engage with the DrugBank curators, to provide a detailed record of changes and to stimulate conversation among the scientific community. Hundreds of excellent user comments have been received and acted on since this feature was implemented. Additionally, we have created a DrugBank Twitter account allowing DrugBank curators to report news, maintenance issues and provide updates, while at the same time allowing users to supply feedback through social media. Engaging our users has always been a priority, and supplying a vehicle to provide feedback will result in higher data quality and an improved user experience.

## CONCLUSION

Over the past 7 years DrugBank has grown from a small, somewhat specialized drug database, to a large, comprehensive drug knowledgebase covering almost all aspects of drug function, formulation and structure. Throughout this evolution, we have strived to maintain the highest quality and currency of data, to continuously improve the quality of its user interface and to attentively listen to all members of its user community. With this latest release of DrugBank, we have continued to make substantial improvements to both the quality/currency of its data and the quality of its user interface. We have also added substantial quantities of new and, what we believe will be, highly relevant data pertaining to drug metabolism, drug metabolites, drug salt-forms, ADMET and QSAR. These additions and enhancements are intended to facilitate research in xenobiotic metabolism (both prediction and characterization), pharmacometabolomics, pharmacokinetics, pharmacodynamics, pharmaceutics and drug design/discovery. They are also intended to make the data within DrugBank relevant to a far wider audience, including researchers in metabolomics, pharmacogenomics and pharmacology, formulation chemists, pharmacists, physicians, educators and the general public. Our commitment to continue to maintain, update and improve DrugBank will continue for the foreseeable future. Based on current trends in pharmaceutical and pharmacological research, it is likely that future versions of DrugBank will include many more drug action and drug metabolism pathways, much more information regarding the transport and uptake of drugs as well as greater detail regarding the molecular mechanisms underlying drug toxicity and drug-associated adverse reactions.

## FUNDING

The authors wish to thank Genome Alberta (a division of Genome Canada), The Canadian Institutes of Health Research (CIHR), and Alberta Innovates Health Solutions (AIHS) for financial support. Funding for open access charge: CIHR.

*Conflict of interest statement.* None declared.
